# Case Report: Zolpidem’s paradoxical restorative action: A case report of functional brain imaging

**DOI:** 10.3389/fnins.2023.1127542

**Published:** 2023-04-14

**Authors:** Jennifer Boisgontier, Kévin Beccaria, Ana Saitovitch, Thomas Blauwblomme, Lelio Guida, Ludovic Fillon, Christelle Dufour, Jacques Grill, Hervé Lemaitre, Stéphanie Puget, Alice Vinçon-Leite, Volodia Dangouloff-Ros, Sarah Charpy, Sandro Benichi, Raphaël Levy, Charles-Joris Roux, David Grévent, Marie Bourgeois, Lila Saidoun, Raphaël Gaillard, Monica Zilbovicius, Nathalie Boddaert

**Affiliations:** ^1^Department of Pediatric Radiology, Necker-Enfants Malades Hospital, AP-HP, Université Paris-Cité, Paris, France; ^2^Imagine Institute, INSERM U1163, Université Paris Cité, Paris, France; ^3^Department of Pediatric Neurosurgery, Necker-Enfants Malades Hospital, AP-HP, Université Paris-Cité, Paris, France; ^4^Department of Pediatric and Adolescent Oncology, Gustave Roussy Institute, Villejuif, France; ^5^Neurodegenerative Diseases Institute, Neurofunctional Imaging Group (GIN), Univ. Bordeaux, CNRS, UMR 5293, Bordeaux, France; ^6^Department of Neurosurgery, Centre Hospitalier Universitaire de Fort de France, University of Antilles, Fort-de-France, Martinique; ^7^Department of Psychiatry, Faculty of Medicine, Sainte-Anne Hospital, Université Paris Cité, Paris, France; ^8^Ecole Normale Supérieure Paris-Saclay, INSERM U1299, ERL “Developmental Trajectories and Psychiatry”: Université Paris Saclay, Université de Paris, CNRS, Centre Borelli, France

**Keywords:** zolpidem, paradoxical effect, neural circuits, brain function, neurological recovery

## Abstract

Zolpidem is a sedative drug that has been shown to induce a paradoxical effect, restoring brain function in wide range of neurological disorders. The underlying functional mechanism of the effect of zolpidem in the brain in clinical improvement is still poorly understood. Thus, we aimed to investigate rest brain function to study zolpidem-induced symptom improvement in a patient who developed postoperative pediatric cerebellar mutism syndrome, a postoperative complication characterized by delayed onset transient mutism/reduced speech that can occur after medulloblastoma resection. The patient experienced clinical recovery after a single dose of zolpidem. Brain function was investigated using arterial spin labeling MRI and resting-state functional MRI. Imaging was performed at three time-points: preoperative, postoperative during symptoms, and after zolpidem intake when the symptoms regressed. Whole brain rest cerebral blood flow (CBF) and resting state functional connectivity using Pearson coefficient correlations between pairs of regions of interest were investigated two-by-two at the different time points. A comparison between postoperative and preoperative images showed a significant decrease in rest CBF in the left supplementary motor area, Broca’s area, and the left striatum and a decrease in functional connectivity within the dentato-thalamo-cortical and cortico-striato-pallido-thalamo-cortical loops. Post-zolpidem images showed increased CBF in the left striatum and increased functional connectivity within the disrupted loops relative to postoperative images. Thus, we observed functional changes within the broader speech network and thalamo-subcortical interactions associated with the paradoxical effect of zolpidem in promoting clinical recovery. This should encourage further functional investigations in the brain to better understand the mechanism of zolpidem in neurological recovery.

## 1. Introduction

Zolpidem is a GABAergic non-benzodiazepine hypnotic agent that is gaining increasing interest because of its intriguing paradoxical effect in restoring brain function in a wide range of brain disorders (Bomalaski et al., 2017) Indeed, despite its being recommended as a first-line treatment for insomnia, a paradoxical effect has been described following zolpidem administration, characterized by transient unexpected “awakening “of patients with consciousness disorders (Whyte and Myers, 2009; Whyte et al., 2014; Kim et al., 2016). Long-term effects induced by zolpidem have also been reported, consisting mostly of long-term speech recovery in aphasia after stroke (Cohen et al., 2004) or akinetic mutism after post-anoxic encephalopathy (Brefel-Courbon et al., 2007) as well as in postoperative cerebellar mutism syndrome (Shyu et al., 2011). Overall, the mechanisms underlying the paradoxical effect of zolpidem, as well as its transient or permanent aspect are still poorly understood. Although there are clinical reports of the paradoxical effect of zolpidem exist in the literature, MRI–based studies are still rare preventing a further understanding of the phenomenon (Arnts et al., 2020; Hao et al., 2022). A current hypothesis is that the paradoxical effect may be due to the action of zolpidem on the GABAergic system, regulating the inhibitory influence on the cortico-striato-pallido-thalamo-cortical loop, especially at the striatum subcortical level. With exception of a few PET and SPECT studies suggesting that the paradoxical effect may involve hypometabolism within the cortico-striato-pallido-thalamo-cortical loop (Brefel-Courbon et al., 2007; Gaillard et al., 2007; Schiff, 2010; Williams et al., 2013), there is little information on the functional brain mechanism involved. In this purpose, resting-state functional connectivity (rs-FC), a non invasive technique can be used to investigate functional connectivity, a measure providing insight into brain networks synchronisation and organisation in normal and pathological conditions (Fox and Raichle, 2007). Thus, rs-FC has been used extensively to highlight dysfunction neuronal synchronisation in a wide of variety of neurological disorders [for review see Lee et al. (2013)]. This may provide a potential biomarker to monitor the effectiveness of treatment strategies. Therefore, rest brain function studies are needed to further understand the functional significance of the effect of zolpidem in clinical improvement, which could help to properly support its therapeutic use in neurological disorders.

One of the clinical contexts in which the use of zolpidem has promoted unexpected recovery is postoperative pediatric cerebellar mutism syndrome (pCMS). pCMS is a severe pediatric postoperative complication that can occur in particular after medulloblastoma (MB) resection. It is characterized by transient mutism or a severe reduction in speech that occurs on average within 48 h after surgery, associated with emotional lability and frequently accompanied by cerebellar motor syndrome (Gudrunardottir et al., 2016). Recovery from pCMS can take several days up to several weeks and long-lasting neurological sequelae, particularly affecting language, may remain (Grill et al., 2004; Paquier et al., 2020). Although there is no consensus concerning the therapeutic approach, clinicians empirically support the use of zolpidem in the context of pCMS. Although the physiopathology of pCMS is not yet well established, in a recent brain imaging study, we observed cerebellar abnormalities within the right dentate nucleus (DN) and cortical abnormalities within the left supplementary motor areas (SMA) associated with pCMS. These results suggest that damage to the dentato-thalamo-cortical loop may be at the core of the development of pCMS (Boisgontier et al., 2021). In addition, the observed hypoperfusion of the pre-SMA, a key region for the initiation of articulatory motor program, may be the first step of the cortical processes involved in mutism.

In the context of an ongoing prospective neuroimaging study investigating the neural basis of pCMS, we report here a rest functional brain imaging case study of a seven year-old child who developed pCMS characterized by the severe loss of spontaneous speech, the loss of motivation, and irritability. A single administration dose of zolpidem promoted clinical recovery within 24 h. The patient underwent full clinical evaluation before and after the surgery, as well as after zolpidem intake. As per protocol, multimodal magnetic resonance imaging (MRI) was performed at three time points: preoperative, immediate postoperative images, during which the patient presented pCMS symptoms and post-zolpidem intake, when pCMS symptoms regressed. We investigated zolpidem-induced changes in brain perfusion using arterial spin labeling (ASL)-MRI by measuring cerebral blood flow (CBF) at rest at each clinical stage. In addition, we investigated resting state functional connectivity (rs-FC) within the dentato-thalamo-cortical loop given the involvement of this loop in pCMS (Morris et al., 2009; Avula et al., 2015; Boisgontier et al., 2021). We further explored rs-FC within the cortico-striato-pallido-thalamo-cortical loop, focusing on subcortical rs-FC between the striatum and internal globus pallidus (GPi), thought to be the targets of the paradoxical effect of zolpidem (Schiff, 2010; Williams et al., 2013). Pre-operative rs-FC and post-zolpidem intake patterns were compared to those an age comparable healthy control group.

## 2. Case presentation

A right-handed seven-year-old boy, presented with signs and symptoms of increased intracranial pressure for several months and, gait disturbance and dysmetria for 1 month. Clinical examination revealed a cerebellar syndrome with multi-directional nystagmus and difficulties in walking with enlargement of the sustentation polygon. No behavioral or speech impairments were noted. An MRI showed a large posterior fossa mass invading the vermis, the lower part of the floor of the fourth ventricle, left cerebellar peduncles, and inducing obstructive hydrocephalus. Thus, endoscopic third ventriculostomy was performed to liberate the cerebrospinal fluid pathways and decrease intracranial pressure. The postoperative course was uneventful. The patient underwent a total resection of the mass through a telovelar approach several days later and histopathological examination revealed a medulloblastoma. The clinical status of this patient was independently assessed by a neurosurgeon (K.B) in the context of the clinical follow-up and a researcher (J.B) in the context of an ongoing prospective study investigating the neural basis of pCMS. Of note, as there is no standardized quantification scale for pCMS symptoms, the described symptomatology of the patient was based on our previously classification (Boisgontier et al., 2021) based on the main symptoms described in the literature (Gudrunardottir et al., 2016). Clinical assessment 1 day after surgery revealed the emergence of symptoms such as the severe loss of spontaneous speech, the loss of motivation, mood changes, irritability and a cerebellar syndrome (hypotonia, dysmetria and left ataxia) which characterizes the diagnosis of pCMS. The patient showed no spontaneous speech but was able to respond to yes/no questions if strongly stimulated. In addition, his parents reported that language comprehension appeared to be preserved. Given the lack of improvement in pCMS symptoms, a single dose of zolpidem at noon at one-fourth of the adult dosage (2.5 mg) (dose for adults = 10 mg) was administered on postoperative day 9 with parental agreement. Twenty-four hours after the zolpidem intake, the patient showed enhanced arousal, became able to communicate spontaneously, was less irritable and “awoke,” highlighting a “paradoxical zolpidem effect.” Indeed, the patient spontaneously produced complete sentences and, was able to name body parts (eyes, nose, mouth, ears). In terms of motor skills, the patient was able to walk, with some assistance at first and independently within the following days. The six-month follow up evaluation showed a strictly normal neurological exam and the patient shows complete recovery at the present time. This is an observational study on the effect of a drug administered within standard clinical procedures. An authorization number (N° EUDRACT: 2019 – A0 1613-54) of the local ethics committee of the French Public Hospitals in agreement with the principles of the Declaration of Helsinki of 1975 (as revised in 1983), was obtained to perform specific preoperative and postoperative (immediate and when symptoms regressed) MRI sequences as per protocol. Written informed consent was obtained from the parents for the pediatric patient.

## 3. Results

We first present the results comparing the whole brain rest CBF and rs-FC analyses between two-by-two time points: preoperative compared to immediate postoperative images; preoperative compared to post-zolpidem intake images; and immediate postoperative compared to post-zolpidem intake images. In addition, we also present results of rs-FC comparing the mean correlation coefficients of the patient for the preoperative and post-zolpidem intake images to the healthy control group (n = 5).

### 3.1. Rest whole brain CBF analyses

**Figure 1:**
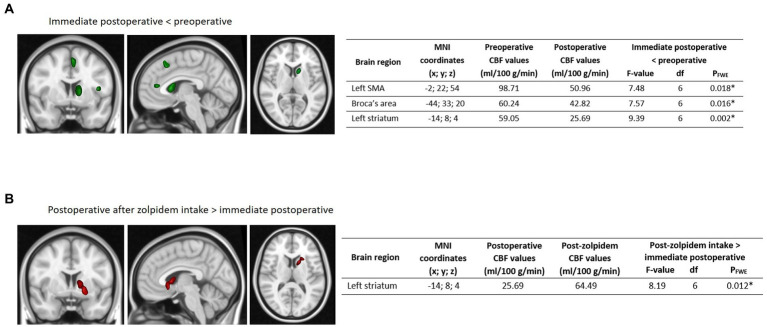
Comparison of whole brain rest CBF at the different time-points. Whole brain results showing **(A)** significantly decreased ASL-CBF values localized in the left SMA, Broca’s area, and left striatum (in green) in the immediate postoperative images relative to the preoperative images and **(B)** significantly increased ASL-CBF values localized in the left striatum (in red) in the post-zolpidem intake images relative to the immediate postoperative images. Maximum intensity projections of significant T statistic clusters were superimposed on a 2D rendering of T1-weighted images in MNI space. The characteristics of significant clusters, with the mean CBF values, are summarized in the respective tables, with results presented using a family-wise error (FWE) correction. MNI refers to the MNI coordinates. R: right, L: left, CBF: cerebral blood flow, SMA: supplementary motor area, ASL: arterial spin labeling, CBF: cerebral blood flow, FWE: family wise error, MNI: Montreal Neurological Institute.

A voxel-wise whole brain comparison of preoperative and immediate postoperative CBF images showed a significant decrease in rest CBF in the left supplementary motor area (SMA) (*t* = 7.48; df = 6 = 4.84; *p* = 0.018) and Broca’s area (*t* = 7.57; df = 6; *p* = 0.016) as well as in one cluster including both the left putamen and in the left caudate constituting together the left striatum (*t* = 9.39; df = 6; *p* = 0.02) in the immediate postoperative images. We also observed a significant increase in rest CBF in the left striatum (*t* = 8.19; df = 6; *p* = 0.012) in the post-zolpidem intake images compared to immediate postoperative CBF images. Finally, there was no significant difference in post-zolpidem intake compared to preoperative CBF images. The corresponding brain regions associated with maximum peak coordinates in the SPM analysis are shown in Figure 1.

### 3.2. Resting state functional connectivity analyses

#### 3.2.1. Comparison of immediate postoperative and preoperative rs-FC images

**Figure 2:**
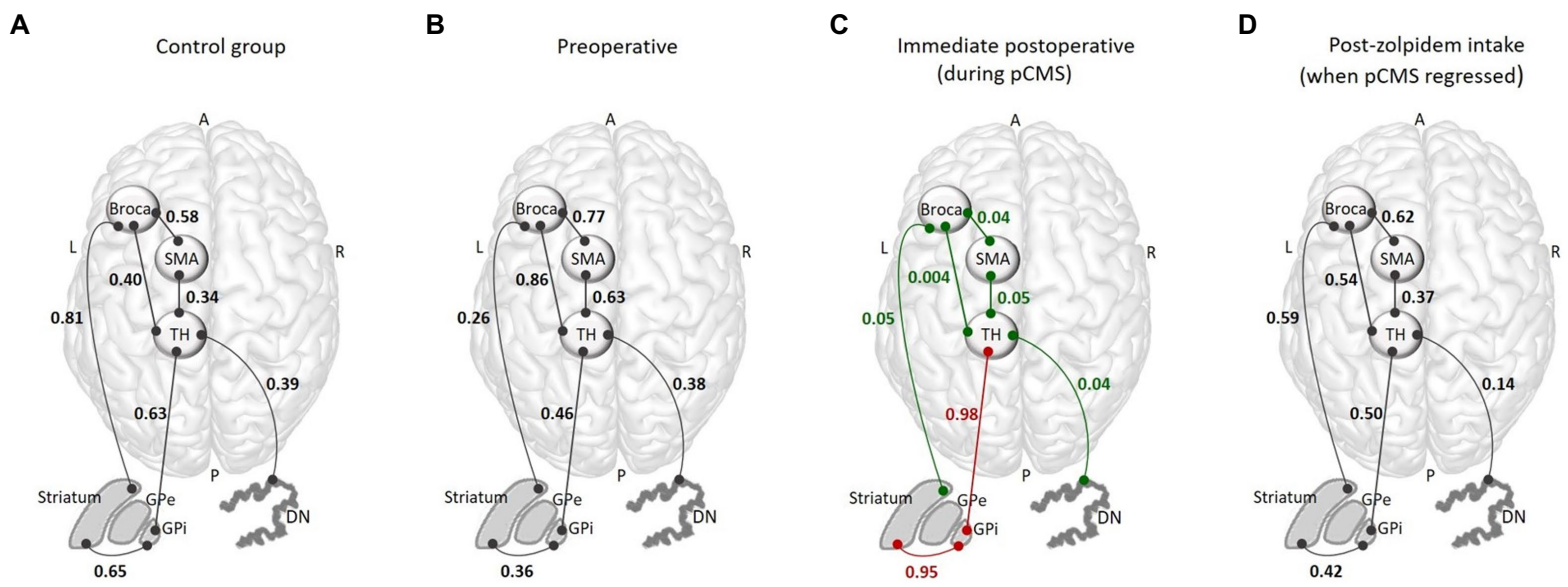
Graphical summary of the FC results within the dentato-thalamo-cortical and cortico-striato-pallido-thalamo-cortical loops for the control group for each time-point. The functional connectivity analysis included the following ROIs: Broca’s area and the left supplementary motor area (SMA), left thalamus, right dentate nucleus (DN), internal globus pallidus (GPi), and left striatum. Altogether, these regions constitute the dentato-thalamo-cortical and cortico-striato-pallido-thalamo-cortical loops. **(A)** Mean of the correlation coefficients obtained for the control group for each pair of ROIs. **(B)** FC correlation coefficients for each pair of ROIs obtained in the preoperative images. **(C)** FC correlation coefficients for each pair of ROIs obtained in the immediate postoperative images, showing decreased (in green) and increased (in red) FC. **(D)** FC correlation coefficients for each pair of ROIs obtained in the post-zolpidem intake, showing a return to the normal pattern. R, right; L, left; A, anterior; P, posterior; pCMS, postoperative cerebellar mutism syndrome; FC, functional connectivity; SMA, supplementary motor area; TH, thalamus; DN, dentate nucleus; GPe, external globus pallidus; GPi, internal globus pallidus; ROIs, regions of interest.

The correlation coefficients were significantly lower in the immediate postoperative images than preoperative images in the following pairs of ROIs: right DN and left t(VL) (*t* = 2.46; df = 6; *p* = 0.049); left t(VA) and left SMA (*t* = 2.60; df = 6; *p* = 0.040); left SMA and Broca’s area (*t* = 2.86; df = 6; *p* = 0.028;); left t(VA) and Broca’s area (*t* = 3.65; df = 6; *p* = 0.014;); and Broca’s area and left striatum (*t* = 2.56; df = 6; *p* = 0.042;). In addition, the correlation coefficients between the left striatum and the left GPi (*t* = −2.94; df = 6; *p* = 0.022) as well as the left GPi and left t(VA) (*t* = −3.14; df = 6; *p* = 0.026) were significantly higher in the immediate postoperative images relative to preoperative (see Figure 2). By contrast, there were no significant differences for any pairs of ROIs in the right hemisphere between the immediate postoperative and preoperative rs-FC images (see Figure 2).

All *p*-values for all statistical comparisons for both hemispheres are shown in Supplementary Table 1.

#### 3.2.2. Comparison of post-zolpidem intake and immediate postoperative rs-FC images

In the post-zolpidem intake images, we observed a significant increase in the correlation coefficients in the following pairs of ROIs: right DN and left t(VL) (*t* = 2.38; df = 6; *p* = 0.048); left t(VA) and left SMA (*t* = 2.48;df = 6; *p* = 0.047); left SMA and Broca’s area (*t* = 2.58; df = 6; *p* = 0.041); left t(VA) and Broca’s area (*t* = 2.49;df = 6; *p* = 0.049); and Broca’s area and left striatum (*t* = 2.36; df = 6; *p* = 0.048) relative to the immediate postoperative images (see Figure 2). No significant differences were observed for any pairs of ROIs in the right hemisphere in post-zolpidem intake images relative to the immediate postoperative images (See Figure 2; Supplementary Table 1).

#### 3.2.3. Comparison of post-zolpidem intake and preoperative rs-FC images

There were no significant differences for any pairs of ROIs in the left hemisphere or right hemisphere between the post-zolpidem intake and preoperative images (see Figure 2; Supplementary Table 1).

#### 3.2.4. Resting-state functional connectivity comparing the patient to the control group

In the preoperative images, there were no significant differences in rs-FC images for the left or right hemisphere between the patient and the control group for any pairs of ROIs. In the post-zolpidem intake images, no significant differences between the patient and the control group were observed for any pairs of ROIs for either hemispheres (see Figure 2; Supplementary Table 1).

## 4. Discussion

To the best of our knowledge, this is the first study to report rest brain perfusion and resting state functional connectivity changes induced by zolpidem in a child who developed pCMS and who quickly experienced symptom improvement. First, the immediate postoperative images showed that the observed severe speech impairment, motivation loss, and emotional lability were associated with a significant decrease in rest CBF in the left supplementary motor area, Broca’s area and the left striatum as well as a decrease in functional connectivity within the dentato-thalamo-cortical and cortico-striato-pallido-thalamo-cortical loops. Second, following zolpidem intake, which induced immediate clinical recovery, we observed an increase in rest CBF in only the left striatum, which may indicate back-to-normal CBF values in Broca’s area and the SMA. In addition, the comparison of post zolpidem and preoperative images showed no significant differences in functional connectivity, suggesting that zolpidem may have restored function within these cortical–subcortical loops. Finally, results showing no significant differences the functional connectivity of the patient and the control group in the preoperative or post-zolpidem intake images suggest that the patient returned to the preoperative rs-FC pattern and that the values returned to normal following zolpidem administration.

Few studies have investigated the effects of zolpidem in brain function. In a PET study, increased metabolism in the orbitofrontal cortex, caudate nucleus and thalamus was observed after zolpidem intake in a patient who presented with apathy (Delorme et al., 2020). The results also showed an increased GABA concentration in the medial frontal cortex and pallidum after zolpidem intake, consistent with the functional changes we described. More recently, Arnts et al. observed a decrease in theta/alpha power and an increase in beta/gamma power over the frontal and parietal cortices in a patient with severe hypoxic–ischemic brain injury after zolpidem intake using EEG and MEG (Arnts et al., 2020). Interestingly, the same EEG pattern was reported for in three patients with severe brain injury who temporary regain conscience after zolpidem intake, suggesting that zolpidem may reduce and restore beta band connectivity through cortical areas (Williams et al., 2013). Together with our findings, these results underlie the major role of the cortico-striato-pallido-thalamo-cortical loop in the paradoxical effect of zolpidem.

In the context of pCMS, our results showing decreased rest CBF in the left SMA and Broca’s area in immediate postoperative ASL images during pCMS are relevant in light of the speech impairments the patient presented, as these cortical areas play a critical role in speech production (La Corte et al., 2021). These results complement our previous findings showing an association between pCMS and left SMA hypoperfusion, which strongly suggest that pCMS may be associated with damage to the ascending cerebellar fibers of the dentato-thalamo-cortical tract (Boisgontier et al., 2021). We also observed a significant decrease in rest CBF within the left striatum in the immediate postoperative relative to the preoperative ASL images. Importantly, the observed hypoperfusion within the left striatum could be secondary, driven by hypoperfusion of the left SMA and Broca’s area caused by dentato-thalamo-cortical tract injury. In addition, our results indicate a widespread reduction in functional connectivity within the left dentato-thalamo-cortical and cortico-striato-pallido-thalamo-cortical loops, further supporting their involvement in pCMS.

Interestingly, while we observed a widespread reduction in functional connectivity within the left dentato-thalamo-cortical loop in the immediate postoperative images, the subcortical functional connectivity between the left striatum and left GPi as well as the left GPi and left t(VA) increased, indicating probable “pathological functional hyperconnectivity.” Although the observed disruption of the cortico-striato-pallido-thalamo-cortical loop may result in subcortical functional hyperconnectivity and may appear to be unexpected, this effect has been previously described in several neurological disorders, such as traumatic brain injury (Amir et al., 2021), multiple sclerosis (Hawellek et al., 2011), cerebrovascular accidents (Nicolo et al., 2015), epilepsy (O’Muircheartaigh et al., 2012), and Parkinson’s disease (Gorges et al., 2015) as well as in psychiatric disorders such as schizophrenia (Park et al., 2021) and bipolar disorder (Guo et al., 2021).

**Figure 3:**
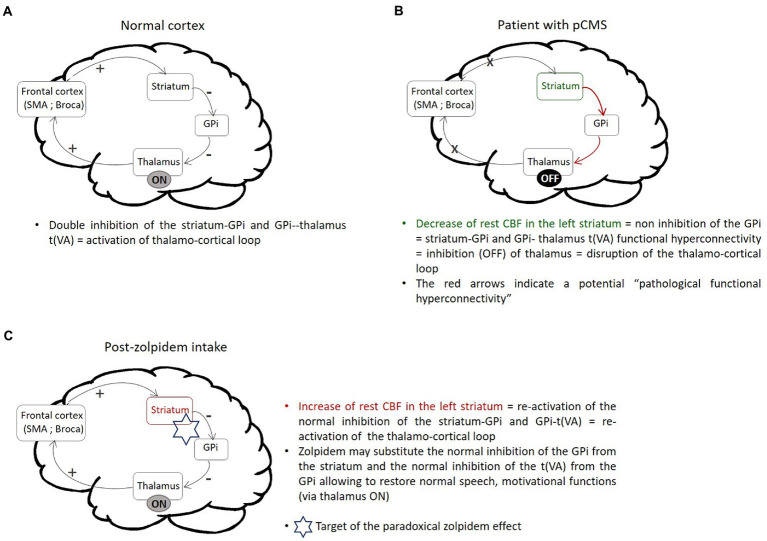
Schematic illustration summarizing the hypothesis for the mechanism of action of the paradoxical effect of zolpidem in pCMS. **(A)** Illustration of the activation of the thalamo-cortical loop in the normal cortex. The frontal cortex activates the striatum. The striatum then inhibits the GPi neurons, which in turn inhibit the t(VA). Such double inhibition from the striatum induces disinhibition (double inhibition = activation) of the thalamus, promoting the excitatory influence of the thalamus on the prefrontal cortices. **(B)** Illustration of the disruption of the thalamo-cortical loop observed in pCMS. The decreased rest CBF observed in the left striatum in the immediate postoperative ASL images may lead to non-inhibition of the GPi, which would affect the connectivity of the striatum-GPi and GPi-t(VA) (“pathological” hyper striatum-GPi and GPi-t(VA) FC) and prevent activation of the thalamo-cortical loop. **(C)** Illustration of the reactivation of the thalamo-cortical loop after zolpidem intake. The increased rest CBF in the left striatum after zolpidem intake may induce reactivation of the normal double inhibition of the GPi from the striatum and the t(VA) from the GPi, therefore re-activating the thalamic influence on the prefrontal cortex, restoring normal speech, motivation, and emotional functions. pCMS, postoperative cerebellar mutism syndrome; SMA, supplementary motor area, globus pallidus; GPi, internal globus pallidus.

An empirical model describing possible targets of the paradoxical effect of zolpidem in cases of certain brain injuries resulting in consciousness disorders, the “mesocircuit model, has been previously proposed (Schiff, 2010). This model implicates the cortico-striato-pallido-thalamo-cortical loop and hypothesizes that zolpidem may substitute the normal inhibition of the GPi from the striatum leading to re-activation of the thalamo-cortical loop in patients with consciousness disorders. Our results suggest that the same mechanism may be involved in the effect of zolpidem in the neural loops involved in pCMS. As illustrated in Figure 3, the disruption of cortical brain networks regulating cognitive functions appears to be reestablished by the action of zolpidem. Importantly, as zolpidem acts on the GABAA a-1 subunit receptor (Crestani et al., 2000) highly expressed in the GPi and striatum (Jin et al., 2011), it may directly inhibit the GPi from the striatum. Thus, we hypothesized that the observed increase in rest CBF in the left striatum after zolpidem intake may reflect re-activation of the normal double inhibition of the GPi from the striatum and the t(VA) from the GPi. Thus, zolpidem may substitute the normal inhibition of the GPi from the striatum which in turn may restore the normal inhibition of the t(VA) from the GPi. This may re-activate the thalamic influence on the prefrontal cortex and subsequently the dentato-thalamo-cortical and cortico-striato-pallido-thalamo-cortical loops, restoring speech, motivation and emotional functions (see Figure 3).

This study had several limitations. Indeed, although it is remarkable to observe such effects on a single patient, these findings are based on a one-patient case study and should therefore be interpreted with caution. Further studies, in particular based on randomized controlled clinical trials on large cohorts are needed to corroborate these results and generalize our understanding of the paradoxical mechanism of action of zolpidem. Moreover, given zolpidem’s pharmacodynamics, immediate and closely spaced assessments could help to further understand this phenomenon.

In conclusion, we believe that our case study presenting brain imaging results helps to extend our understanding concerning the brain mechanism underlying the long-term effect of zolpidem in clinical improvement and highlights the importance of further brain functional studies. Finally, future multicenter and multinational clinical trials, including a large dataset, are still needed to further investigate the paradoxical effect of zolpidem in various clinical contexts (pCMS, aphasia, severe traumatic brain injuries, minimally conscious state) and provide further insight into the mechanisms involved in the effect of zolpidem in neurological recovery.

## Data availability statement

The original contributions presented in the study are included in the article/Supplementary material, further inquiries can be directed to the corresponding author.

## Ethics statement

The studies involving human participants were reviewed and approved by French Public Hospital: Ethic Commitee “Sud-Est II.” Written informed consent to participate in this study was provided by the participants’ legal guardian/next of kin. Written informed consent was obtained from the individual(s), and minor(s)’ legal guardian/next of kin, for the publication of any potentially identifiable images or data included in this article.

## Author contributions

JB, KB, and NB performed data collection. JB and LF performed the image preprocessing. JB, AS, HL, AV-L, and NB performed the statistical analyses. KB, VD-R, RL, C-JR, and DG assisted in the data acquisition. JB, KB, TB, LG, CD, JG, SP, SB, MB, RG, MZ, and NB provided the clinical relevance context. JB, KB, AS, TB, LF, LG, CD, JG, HL, SC, SP, SB, MB, RG, MZ, and NB interpreted the results. KB, AS, TB, LF, LG, CD, JG, HL, SC, SP, SB, AV-L, VD-R, RL, C-JR, RG, MZ, and NB revised the paper. JB and AS generated the figures and tables. JB, KB, AS, MZ, and NB wrote the paper. All authors contributed to the article and approved the submitted version.

## Funding

This work was supported in part by Lisa Forever association (https://www.lisaforever.org/), Canceropole “Ile de France” (https://www.canceropole-idf.fr/), Institut National du Cancer (InCa), Fondation de l’avenir (https://www.fondationdelavenir.org/), and Fondation ARC pour la recherché sur le cancer (https://www.fondation-arc.org/).

## Conflict of interest

The authors declare that the research was conducted in the absence of any commercial or financial relationships that could be construed as a potential conflict of interest.

## Publisher’s note

All claims expressed in this article are solely those of the authors and do not necessarily represent those of their affiliated organizations, or those of the publisher, the editors and the reviewers. Any product that may be evaluated in this article, or claim that may be made by its manufacturer, is not guaranteed or endorsed by the publisher.

## Glossary

ASL: arterial spin labeling
CBF: cerebral blood flow
DN: dentate nucleus
FC: functional connectivity
GPi: internal globus pallidus
L: left
MB: medulloblastoma
MNI: Montreal Neurological Institute
MRI: magnetic resonance imaging
pCMS: postoperative cerebellar mutism syndrome
ROI: region of interest
R: right
rs-FC: Resting state functional connectivity
SMA: supplementary motor area
VA: ventral anterior
VL: ventral lateral
