# Supplementary material for: Case Report: Zolpidem’s paradoxical restorative action: A case report of functional brain imaging
Source: Front Neurosci. 2023 Apr 14;17:1127542. doi: 10.3389/fnins.2023.1127542 (PMC10140395; doi:10.3389/fnins.2023.1127542)
Supplement: Supplementary file 2 [file Data_Sheet_1.pdf]

Article Title : Zolpidem's paradoxical restorative action : a case report of functional brain imaging

Jennifer Boisgontier*[1,2], Kévin Beccaria[3], Ana Saitovitch[1,2], Thomas Blauwblomme[3], Lelio Guida[3], Ludovic Fillon[1,2], Christelle Dufour[4], Jacques Grill[4], Hervé Lemaitre[5], Stéphanie Puget[6], Alice Vinçon-Leite[1,2], Volodia Dangouloff-Ros[1], Sarah Charpy[1], Sandro Benichi[3], Raphaël Levy[1], Charles-Joris Roux[1], David Grévent[1], Marie Bourgeois[3], Lila Saidoun[4], Raphaël Gaillard[7], Monica Zilbovicius[8], and Nathalie Boddaert[1,2]

***** Corresponding Author : Jennifer Boisgontier

Supplementary Material 1

Imaging

MRI data acquisition: Apart from the sequences in the clinical context for tumor evaluation, the patient underwent MRI scans including 3D T1-weighted sequences (156 axial slices; TR/TE=6.9/3 ms; resolution in plane = 1 × 1 mm, slice thickness = 1 mm, voxel size = 1 × 1 × 1 mm^3, duration = 6 min); pseudo-continuous perfusion ASL-MRI sequences for measures of rest CBF using a fast spin echo acquisition (TR/TE = 4453/10.96 ms, number of axial slices = 40, resolution in plane = 1.875 × 1.875 mm, slice thickness = 4 mm, voxel size = 1.875 × 1.875 × 4 mm^3 ; post-labelling delay (PLD) = 1025 ms, labelling duration = 1500 ms; duration = 4 min), and resting-state functional MRI sequences: TR/TE = 2500/31 ms, number of axial slices = 45, resolution in plane = 3.125 × 3.125 mm, slice thickness = 3 mm, voxel size = 3.125 × 3.125 × 3 mm^3, duration =10 min (240 volumes). All images were acquired at three time points: preoperative (6 days prior to surgery), immediate postoperative (1 day after surgery) and post-zolpidem intake (1 day after the single dose of zolpidem intake). Sequences similar to those of the patient were obtained for five healthy children of comparable for age to constitute a control group. All images were obtained using the same 3 Tesla MRI scanner (Discovery MR 750 General Electric).

Preprocessing

ASL perfusion imaging: All ASL-MRI images were preprocessed using Statistical Parametric Mapping 12 (https://www.fil.ion.ucl.ac.uk/spm/software/spm12/). After visually checking for major artefacts, 3D T1-weighted images were segmented into grey matter, white matter and cerebrospinal fluid to obtain deformation fields between native space and Montreal Neurological Institute (MNI) space. Then, ASL-MRI images were co-registered to their native grey matter images. Using the deformation field obtained from the normalization, co-registered ASL-MRI images were spatially normalized into MNI space. Finally, the resulting images were smoothed using a Gaussian kernel of 6 mm full-width at half-maximum. The quality of the co-registred ASL-MRI images were visually inspected by one imaging processing expert (LF) to perform a voxel-wise, whole brain approach.

Resting-state functional imaging: Following the pipeline of the Data Processing Assistant for Resting-State functional MRI (DPARSFA) toolbox (http://rfmri.org/DPARSF), all resting-state functional images were preprocessed as follows. First, the 10 first volumes were removed for the signal stabilization. Then, a correction for slice timing acquisition and one for head motion using a six-parameter (rigid body) linear transformation (< 0.2mm) were applied. We co-registered individual T1-weighted images to the mean functional image. Co-registered images were segmented into six tissue types (white matter, grey matter, cerebrospinal fluid, bone and air) and normalized into MNI space (voxel size = 3mm) using the DARTEL tool (Ashburner, 2007). In addition, the nuisance regressor included the white matter and cerebrospinal fluid signal to reduce respiratory and cardiac effects as well as the Friston 24-parameter model (Friston et al., 1996) to regress out head motion effects. Next, the functional images were temporally band-pass filtered at 0.01–0.1 Hz. In the final step, functional images were smoothed using a 6mm isotropic Gaussian filter. After visually checking the quality of the normalization process, the smoothed and normalized images were used to carry out the rs-FC analyses based on regions of interest (ROIs)

Resting-state functional connectivity analyses

Definition of ROIs: rs-FC was assessed with a ROI approach using existing anatomical atlases in MNI space. All pairs of ROIs, coherently anatomically and functionally connected within the dentato-thalamo-cortical and cortico-striato-pallido-thalamo-cortical loops were defined bilaterally. We used the WFU PickAtlas (https://www.nitrc.org/projects/wfu_pickatlas/) to define the bilateral DN as well as the automated anatomical labelling atlas 3 (AAL3) (https://www.gin.cnrs.fr/fr/outils/aal/) to define bilaterally the supplementary motor area (SMA), the thalamic nuclei and the triangularis and opercularis pars of the left inferior frontal gyrus. We formed Broca's area by merging the triangularis and opercularis pars of the left inferior frontal gyrus. The GPi and the striatum were defined using an atlas on the basal ganglia (https://www.nitrc.org/projects/atag/). We identified the thalami nuclei anatomically connected with the defined ROIs: ventral anterior t(VA) and ventral lateral t(VL). The final pairs of ROIs with the corresponding thalamic nuclei, bilaterally, were as follows: DN and t(VL); t(VA) and SMA; GPi and the t(VA), inferior frontal gyrus (triangularis and opercularis pars) and t(VA) for right hemisphere and the t(VA) and Broca's area for left hemisphere.

Statistical analyses

Rest CBF whole brain analyses: ASL-MRI, whole brain voxel-wise comparisons were performed using SPM12 to examine differences between the preoperative and immediate postoperative, immediate postoperative images and, the post-zolpidem intake as well as the post-zolpidem intake images and preoperative. Two-by-two comparisons for the different time points were performed within the framework of a general linear model using one-way ANOVA. To allow the estimation of inter-individual variability, the control group was included in the statistical model, as used in other studies (Pesenti et al., 2001; Boddaert et al., 2005) and recommended in (Moser, 2020). Voxels were considered to be significant for $p < 0.05$, with a family-wise-error (FWE) correction for multiple comparisons.

Resting-state functional connectivity analyses: Using the DPARSFA toolbox, we computed Pearson correlation coefficients for each respective pair of ROIs for all participants (patient and controls). rs-FC comparisons were performed using R version 4.0.2 (https://www.r-project.org/) to examine differences between preoperative and immediate postoperative, immediate postoperative and post-zolpidem intake and post-zolpidem intake and preoperative images. The same statistical design as the

ASL-MRI analyses was used. In addition, a comparison between preoperative and post-zolpidem intake and the mean value of each pair of ROIs for the control group were carried out using one-way ANOVA to study the preoperative rs-FC pattern, as well as potential to back-to-normal FC values. The significance threshold was set to 0.05 and we corrected p-values for multiple comparisons using the Benjamini-Hochberg false discovery rate procedure (Benjamini and Hochberg, 1995).
